# Evaluation of Technical Issues in a Pilot Multicenter Newborn Screening Program for Sickle Cell Disease

**DOI:** 10.3390/ijns5010002

**Published:** 2018-12-21

**Authors:** Maddalena Martella, Giampietro Viola, Silvia Azzena, Sara Schiavon, Andrea Biondi, Giuseppe Basso, Paola Corti, Raffaella Colombatti, Nicoletta Masera, Laura Sainati

**Affiliations:** 1 Dipartimento di Salute della Donna e del Bambino, Università di Padova, 35128 Padova, Italy; 2 Dipartimento di Pediatria, Università di Milano-Bicocca-Fondazione MBBM, San Gerardo Hospital, 20900 Monza, Italy

**Keywords:** sickle cell disease, high performance liquid chromatography (HPLC), β-globin gene

## Abstract

A multicenter pilot program for universal newborn screening of Sickle cell disease (SCD) was conducted in two centres of Northern Italy (Padova and Monza). High Performance Liquid Chromatography (HPLC) was performed as the first test on samples collected on Guthrie cards and molecular analysis of the β-globin gene (*HBB*) was the confirmatory test performed on the HPLC-positive or indeterminate samples. 5466 samples of newborns were evaluated. Of these, 5439/5466 were submitted to HPLC analysis and the molecular analysis always confirmed in all the alteration detected in HPLC (62/5439 newborns); 4/5439 (0.07%) were SCD affected, 37/5439 (0.68%) were HbAS carriers and 21/5439 (0.40%) showed other hemoglobinopathies. Stored dried blood spots were adequate for HPLC and β-globin gene molecular analysis. Samples were suitable for analysis until sixteen months old. A cut-off of A_1_ percentage, in order to avoid false negative or unnecessary confirmation tests, was identified. Our experience showed that several technical issues need to be addressed and resolved while developing a multicenter NBS program for SCD in a country where there is no national neonatal screening (NBS) program for SCD and NBS programs occur on a regional basis.

## 1. Introduction

Neonatal screening (NBS) for Sickle cell disease (SCD) is an effective tool for the early detection of affected individuals, to direct them at the clinical programs to prevent complications and finally to offer genetic counseling to a family and is therefore highly recommended as the first step of comprehensive care [1,2,3,4,5,6,7]. In detail, early identification of affected SCD patients through a NBS program allows the introduction of penicillin prophylaxis from two months of age and performing of an adequate vaccination schedule with the reduction of mortality from infection, prompt enrolment in comprehensive care programs with timely parent health education, Trans Cranial Doppler (TCD) screening and prevention of acute events [1,2].

In Italy a national NBS program is not available and NBS programs are organized on a regional basis [8]. Haemoglobinopathies are included in the regional NBS program of only 1 out of the 20 regions [9], but some pilot programs have been conducted in the past years [10,11,12].

Padova and Monza, two towns located in the North of Italy, in the Veneto and Lombardia Regions respectively, have a high percentage of an immigrant population and an average of more than 3500 births every year, with 25% of them from immigrant parents [13]. We developed a pilot multicenter, multiregional universal NBS program for SCD [14].

The methods used and recommended for the SCD neonatal screening by International Guidelines can be qualitative, as IsoElectric Focusing (IEF), and quantitative, as Capillary Electrophoresis (CE), High Performance Liquid Chromatography (HPLC) and recently the Tandem Mass Spectrometry (MS/MS) [15], but the abnormal chromatogram must always have a confirmatory test with the different methodologies. Our protocol is an innovative way for SCD screening with the combination of HPLC analysis and molecular analysis. This type of approach has been used in very few studies [16,17].

**Figure 1:**
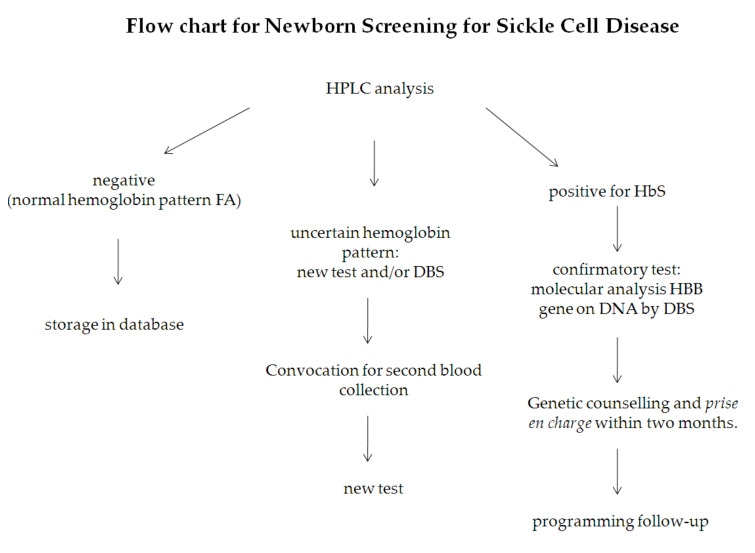
Flow chart for newborn screening for Sickle cell disease.

The purpose of this manuscript is to highlight some of the technical challenges that we had to face in the development of our pilot screening. The objectives of this study were: (i) to define the sensitivity of our methods in order to establish a cut-off percentage for normality in the presence of low levels of β-globin chains, (ii) to maintain high-quality results to ensure the accurate interpretation of the analysis for immediate initiation of supportive care for affected newborns, (iii) to check the stability of the sample over time to ensure reliable measurements, a very important issue in a multicenter setting, (iv) to verify the organization and feasibility of a multiregional screening program. Finally, the flow chart for newborn screening for SCD is shown in Figure 1.

## 2. Materials and Methods

### 2.1. Study Design and Population

In Padova the NBS pilot program enrolled newborns from 2 May 2016 until 30 November 2017 while in Monza from 1 September 2016 until 31 August 2017. The screening was performed on all newborns from the 36th hour to the third day after birth. Blood collection was performed by pricking the heel of the newborn and collection on a Guthrie card (903 Whatman^®^ in Padova and AHLSTROM^®^ 226 in Monza) after informed consent from the parents was obtained.

Dried blood spots from Monza were sent to Padova through courier once a week and stored at room temperature and in a dark dry place in the NBS laboratory of Padova’s Clinic of Pediatric Hematology-Oncology. The samples were analyzed after 7 days and the first test was performed with the VARIANT NBS Newborn Screening System (Bio-Rad Laboratories, Munich, Germany) and the confirmatory test, on the same sample, with molecular analysis of β-globin gene.

### 2.2. High Performance Liquid Chromatography (HPLC)

All specimens were first examined by High Performance Liquid Chromatography (HPLC) performed on a VARIANT NBS Newborn Screening System (Bio-Rad Laboratories) following the manufacturer’s recommendations, within 7 days after blood spots had been taken. All chromatograms were automatically analyzed and visually inspected for absent Hemoglobin A and variant hemoglobins. The columns and all reagents such as buffers, primers, and hemoglobin standards were purchased from the manufacturer. Two or three disks (diameter of 3.2 mm) were punched out of the dried blood spot (DBS) and placed in a well of 96-well plate round bottom. In each well 280µL of distilled water was added and mixed with the pipette several times at room temperature. Two mixtures of hemoglobin standards FAES and FADC, respectively were analyzed in duplicate even if the run included more plates.

HPLC uses an ion exchange column with gradient elution. The presence of different hemoglobins is revealed through a UV-VIS detector settled at 415 nm. The time that passes from the injection of the sample to the output of the peak of hemoglobin type, is known as the retention time of that particular hemoglobin and represents a reproducible value for that column, at that gradient elution and at that temperature. For the different hemoglobins we have different retention times and characteristic chromatographic profiles, with the exception of HbE and HbA_2_ that elute in the same peak, therefore making them indistinguishable from each other.

In addition, it is possible to quantify in a relative way the percentage of the different hemoglobins; the HPLC analysis helps to identify the differences between carrier individuals (HbS/HbA) and homozygous affected individuals (HbS/HbS) and also to differentiate heterozygous compounds such as HbS/HbC.

Moreover, the sensibility of the protocol has been verified with experiments of dilution on the SCD sample (HbSS) until 1:10.

### 2.3. Molecular Analysis of the β-globin Gene

Molecular analysis of the β-globin gene (*HBB*; MIM # 603903; NM_000518) represented the confirmatory test and was performed on all the following samples: those with an altered hemoglobin profile at HPLC, due to the presence of HbS or another hemoglobin variant; the specimens with HbA<5% or with HbA>30%, excluded transfusion anamnesis, were sent for molecular study. The samples with a red blood cell transfusion and HbA>30% were submitted for HPLC analysis three months later.

Genomic DNA, extracted using the Qiagen kit (QIAamp^®^DNA Mini and Blood), from blood spots allowed to air dry, were amplified by chain reaction of polymerase (PCR) at the exon level and at the intron-exon junctions with the primers below reported: *HBB1* Fw5′-AAAAGTCAGGGCAGAGCCAT-3′, *HBB1* Rw5′-CCCAGTTTCTATTGGTCTCCTTAA-3′, *HBB2* Fw5′-GGGTTTCTGATAGGCACTGACTC-3′, *HBB2* Rw5′-AAAAGAAGGGGAAAGAAAACATCA-3′, *HBB3* Fw5′-TAGCAGCTACAATCCAGCTACCA-3′, *HBB3* Rw5′-GGACTTAGGGAACAAAGGACCT-3′.

Alterations of the coding sequence were analyzed and characterized by sequencing of the hemoglobin β chain coding *HBB* gene using an ABI Prism^®^ 310 Genetic Analyzer Applied Biosystems. Sanger sequencing is the most comprehensive method of mutation detection and determines the exact sequence spanning the area of the primers used.

### 2.4. Management of Results

HPLC negative samples for abnormal hemoglobins didn’t require further analysis. While, HPLC positive samples for abnormal chains underwent a confirmatory test with molecular analysis of the β-globin gene on the same sample collected at the nursery. Families with SCD children were called for a visit in the clinic within two months, while parents of HbS carriers were called for a visit and counselling within six months. Carriers of other hemoglobin variants received a letter, informing them of the results.

## 3. Results

In this study, 5466 samples of newborns from both birth centers were enrolled. Of these, 5439/5466 (2821/2826 of Padova, 99.8% and 2618/2640 of Monza 99.1%) were submitted for HPLC analysis. None of the samples collected were excluded from the analysis. Each sample was analyzed within 7 days after the blood had been spotted on the paper.

The results are summarized in Table 1. Molecular analysis always confirmed the abnormality detected in HPLC in 62/5466 newborns; other hemoglobin variants were also detected (HbC, HbD, HbE) in 0.5% of the cases in Monza and in 0.21% of those in Padova.

**Figure 2:**
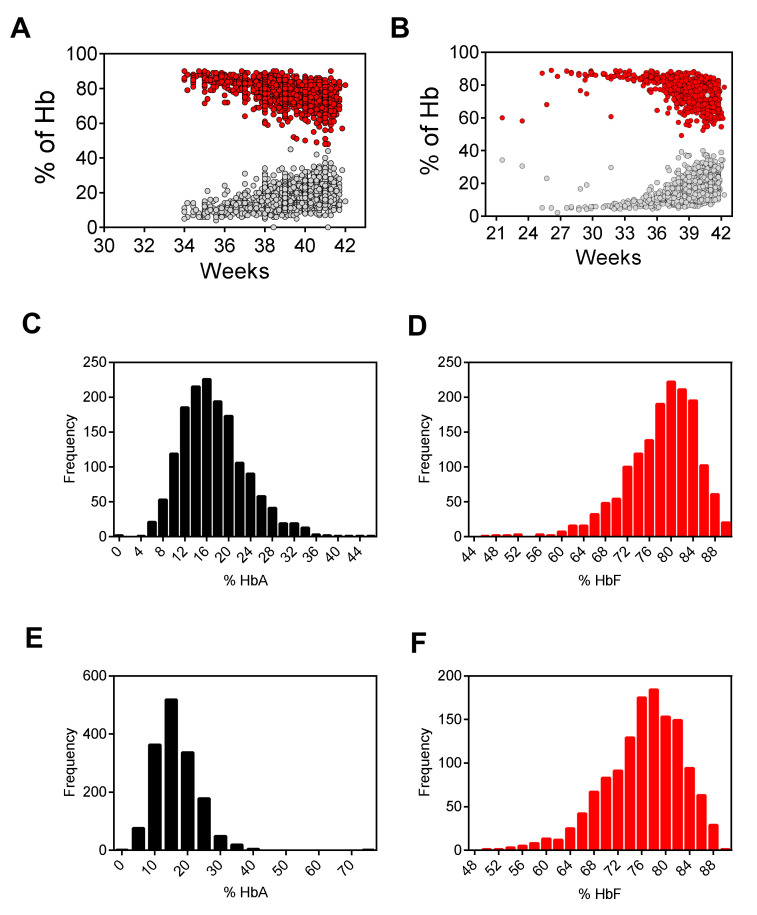
Percentages of HbF (red circles) and HbA (gray circles) according to the gestational age (weeks from conception to blood withdrawal) found in the cohort of Padova (**A**) and Monza (**B**). Distribution frequency of HbA and HbF in the cohort of Padova (**C**,**D**) and Monza (**E**,**F**).

28 samples (0.86%) of preterm infants, showed a value of HbA<5% at HPLC. Molecular analysis confirmed that 26/28 had genotype HbAA, while 2 samples with HbA values of 2.2% and 3.8%, respectively, had a genotype HbAD and a FAST value >10% (potential HbBart) and had been reported to the referring center. In an attempt to determine normal values for HbA and total HbF, the peak percentages of samples with a normal hemoglobin pattern were plotted against the gestational week. The results (see Figure 2) showed a correct Gaussian distribution for the HbA and HbF with a mean of 16.6% and 77.1% respectively as expected for at term newborns.

All dried blood spot cards collected and the storage methods turned out adequate both for HPLC analysis and β globin gene molecular analysis.

The experiments of dilution on the SCD sample (HbSS) detected low levels of HbS until 1:10.

**Figure 3:**
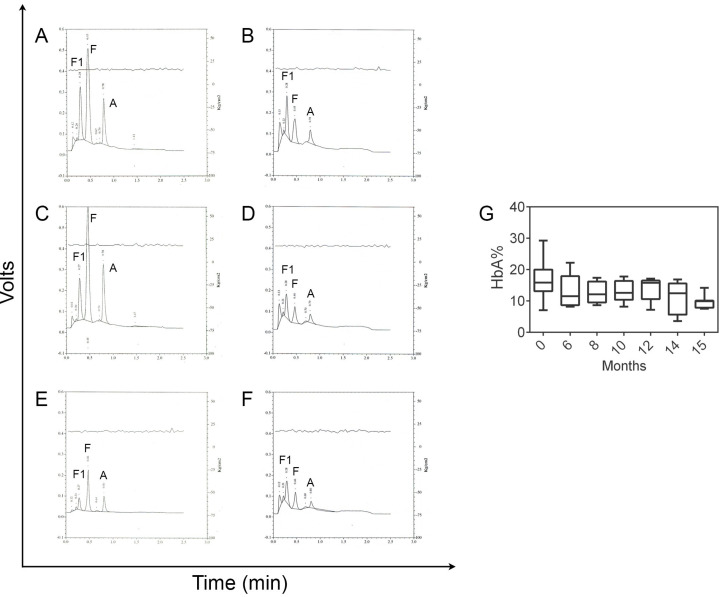
Representative chromatograms of a dried blood spot analyzed within 7 days from blood withdrawal (**A**,**C**,**E**) and after four (**B**), eight (**D**) and sixteen months (**F**). Percentages of HbA analyzed after different months from the blood withdrawal (**G**). At least five samples for group were analyzed.

In our experience we verified storage conditions and the reliability of the DBS a few months later [16] to understand if it was possible to identify the different hemoglobins according to our standard settings of the instrument. We repeated the HPLC analysis of different DBS series stored for sixteen months. In each series, the values of HbA peak area percentage identified in HPLC analysis, although reduced, allowed for the detection of the hemoglobin peaks; retention time was not modified by the samples aging: the HbA value was never <7% and never <800,000 µV*s, in terms of area under the curve (AUC) according to manufacturer instructions. HbS, when present was easily identified by HPLC analysis and similarly to HbA it was not influenced by sample aging [18]. The reduction of the HbA percentage should be attributed to physiological degradation of HbA and to the presence of cellulose particles removed from the Guthrie card together with the blood. In this study, the DBS up to sixteen months old provided satisfactory results (see Figure 3). The quality and the efficiency of the analysis was not affected by different types of Guthrie cards and the two different types of Guthrie cards gave the same performance.

Furthermore, we have noted in our experience that the needle restrains randomly, probably by electrostatic reasons, some blood spots that could cause not only the obstruction of the needle, but also the contamination of other wells. Thus, we recommend that the run must be always monitored and if necessary the run should be interrupted. At the end of every analysis session the instrument must be carefully cleaned.

## 4. Discussion

Our study represents the first multicenter pilot project of universal NBS for SCD in two different regions of Northern Italy.

The methods of investigation were adequate and highly specific, the confirmatory test always confirmed the abnormal hemoglobinopathies detected in HPLC. The two different types of Guthrie cards gave the same performances. The quality, the storage of the sample and the volume of the collected blood are very important factors that may affect the results in particular the retention time in HPLC of the different hemoglobins [19]. Our setting proved to be effective.

Moreover, in our experience we noted that it is very important for the setting of the 96-well plate for HPLC analysis to ensure the acquisition of the total area >800,000 µV*s, as recommended by the manufacture: The number of the spots, the volume of the water for Hb elution, mixed with the pipette several times at room temperature. The 96-well plate can be store at room temperature. This protocol ensures a homogeneous solution to detect the different hemoglobins. The sensitivity of the system can be considered optimal for all different hemoglobins in agreement with the hemoglobin standards FAES and FADC provided by the manufacturer. It is worthwhile to note that our data showed a normal distribution both for HbA and HbF in well agreement to that reported by Bouva et al. [20].

Finally, the sensibility of the protocol verified with experiments of dilution on the SCD sample (HbSS) until 1:10 guaranteed the possibility ofdetecting low levels of HbS.

Newborns born prematurely may yield misleading results. Premature infants may have very low levels of A_1_ hemoglobin at birth; in the absence of a significant amount of A_1_, β chains mutations can be undetectable. The cut-off of HbA<5% is efficient also for preterm newborns. The percentages of HbA and HbF were in agreement with previous reports [21,22], which found a great variability according to the maternal ethnic origin and the gestational age.

The VARIANT NBS Newborn Screening System for our experience has critical issues: the spots can remain attached to the needle causing obstruction or even the random fall of the spot. Therefore, caution is necessary and it is better to check the run and to wash frequently the needle and the instrument.

## 5. Conclusions

Our data demonstrate the feasibility of a multicentric SCD screening and indicate the robustness and reliability of the screening system. The data obtained from HPLC analysis were in excellent agreement with the large amount of data present in the literature. The sensitivity of the system can be considered optimal in conjunction with the molecular analysis of the β globin gene. A scaling up of the project to other areas of the two regions is now planned.

## Figures and Tables

**Table 1:** Hemoglobin patterns observed in the pilot newborn screening.

Hb Pattern (HPLC)	HBB Genotype	Newborns (n.)
FAS	*HBB:* c.20A>T/wt	37 (0.68%)
FS	*HBB:* c.20A>T/c.20A>T	3 (0.055%)
FSC	*HBB:* c.20A>T/c.19A>T	1 (0.02%)
FAC	*HBB:* c.19A>T/wt	9 (0.16%)
FAD	*HBB:* c.364G>C/wt	8 (0.15%)
FAE	*HBB:* c.79C>T/wt	4 (0.07%)
Total		62 (1.14%)

n. = number of newborns.

## References

[1] Ware R.E., de Montalembert M., Tshilolo L., Abboud M.R. (2017). Sickle cell disease. Lancet.

[2] Piel F.B., Steinberg M.H., Rees D.C. (2017). Sickle Cell Disease. N. Engl. J. Med..

[3] National Health System (NHS) Sicke Cell Disease in Childhood. https://assets.publishing.service.gov.uk/government/uploads/system/uploads/attachment_data/file/408961/1332-SC-Clinical-Standards-WEB.pdf.

[4] De Montalembert M., Ferster A., Colombatti R., Rees D.C., Gulbis B. (2011). European Network for Rare and Congenital Anemias. ENERCA clinical recommendations for disease management and prevention of complications of sickle cell disease in children. Am J. Hematol..

[5] Colombatti R., Perrotta S., Samperi P., Casale M., Masera N., Palazzi G., Sainati L., Russo G., Italian Association of Pediatric Hematology-Oncology (AIEOP) Sickle Cell Disease Working Group (2013). Organizing national responses for rare blood disorders: The Italian experience with sickle cell disease in childhood. Orphanet J. Rare Dis..

[6] De Franceschi L.R.G., Sainati L., Venturelli D. SITE-AIEOP Recommendations for Sickle Cell Disease Neonatal Screening. http://www.site-italia.org/collana_scientifica.php.

[7] Lobitz S., Telfer P., Cela E., Allaf B., Angastiniotis M., Backman J.C., Badens C., Bento C., Bouva M.J., Canatan D. (2018). Newborn screening for sickle cell disease in Europe: Recommendations from a Pan-European Consensus Conference. Br. J. Haematol..

[8] http://www.aismme.org.

[9] De Zen L., Dall’Amico R., Sainati L., Colombatti R., Testa E.R., Catapano R., Zanolli F. Screening neonatale per le emoglobinopatie su Dried Blood Spot. Proceedings of the XXXVI Congresso Nazionale Associazione Italiana Ematologia Oncologia Pediatrica (AIEOP).

[10] Rolla R., Castagno M., Zaffaroni M., Grigollo B., Colombo S., Piccotti S., Dellora C., Bona G., Bellomo G. (2014). Neonatal screening for sickle cell disease and other hemoglobinopathies in “the changing Europe”. Clin. Lab..

[11] Venturelli D., Lodi M., Palazzi G., Bergonzini G., Doretto G., Zini A., Monica C., Cano M.C., Ilaria M., Montagnani G. (2014). Sickle cell disease in areas of immigration of high-risk populations: A low cost and reproducible method of screening in northern Italy. Blood Transfus..

[12] Ballardini E., Tarocco A., Marsella M., Bernardoni R., Carandina G., Melandri C., Guerra G., Patella A., Zucchelli M., Ferlini A. (2013). Universal neonatal screening for sickle cell disease and other haemoglobinopathies in Ferrara, Italy. Blood Transfus..

[13] (2017). RapportoAnnuale ISTAT. https://www.istat.it/it/files//2017/05/RapportoAnnuale2017.pdf.

[14] Martella M., Cattaneo L., Viola G., Azzena S., Cappellari A., Baraldi E., Zorloni C., Masera N., Biondi A., Basso G. Universal Newborn Screening for Sickle Cell Disease: Preliminary Results of the First Year of a Multicentric Italian Project. Proceedings of the 22nd Annual Congress of the European Hematology Association.

[15] Lobitz S., Klein J., Brose A., Blankenstein O., Frömmel C. (2017). Newborn screening by tandem mass spectrometry confirms the high prevalence of sickle cell disease among German newborns. Ann. Hematol..

[16] Detemmerman L., Olivier S., Bours V., Boemer F. (2017). Innovative PCR without DNA extraction for African sickle cell disease diagnosis. Hematology.

[17] Kunz J.B., Awad S., Happich M., Muckenthaler L., Lindner M., Gramer G., Okun J.G., Hoffmann G.F., Bruckner T., Muckenthaler M.U. (2016). Significant prevalence of sickle cell disease in Southwest Germany: Results from a birth cohort study indicate the necessity for newborn screening. Ann. Hematol..

[18] Martella M., Viola G. (2018). Chromatograms derived from HPLC analysis.

[19] Frömmel C., Brose A., Klein J., Blankenstein O., Lobitz S. (2014). Newborn Screening for Sickle Cell Disease: Technical and Legal Aspects of a German Pilot Study with 38,220 Participants. BioMed Res. Int..

[20] Bouva M.J., Mohrmann K., Brinkman H.B.J.M., Kemper-Proper E.A., Elvers B., Loeber J.G., Verheul F.E.A.M., Giordano P.C. (2010). Implementing neonatal screening for haemoglobinopathies in the Netherlands. J. Med. Screen..

[21] CorteÂs-Castell E., PalazoÂn-Bru A., Pla C., Goicoechea M., Rizo-Baeza M.M., Juste M., Gil-Guillen V.F. (2017). Impact of prematurity and immigration on neonatal screening for sickle cell disease. PLoS ONE.

[22] Allaf B., Patin F., Elion J., Couque N. (2018). New approach to accurate interpretation of sickle cell disease newborn screening by applying multiple of median cutoffs and ratios. Pediatr. Blood Cancer.

